# Socio-demographic correlates of diabetes self-reporting validity: a study on the adult Kurdish population

**DOI:** 10.1186/s12902-022-01056-w

**Published:** 2022-05-26

**Authors:** Farhad Moradpour, Negar Piri, Hojat Dehghanbanadaki, Ghobad Moradi, Mahdiyeh Fotouk-Kiai, Yousef Moradi

**Affiliations:** 1 Social Determinants of Health Research Center, Research Institute for Health Development, Kurdistan University of Medical Sciences, Sanandaj, Iran; 2 Health Network of Dehgolan, Kurdistan University of Medical Sciences, Sanandaj, Iran; 3 Endocrinology and Metabolism Research Center, Endocrinology and Metabolism Clinical Sciences Institute, Tehran University of Medical Sciences, Tehran, Iran; 4 Department of Epidemiology and Biostatistics, Faculty of Medicine, School of Medicine, Kurdistan University of Medical Sciences, Sanandaj, Iran; 5 Endocrinology & Metabolism, Department of Internal Medicine, School of Medicine, Tohid Hospital, Kurdistan University of Medical Sciences, Sanandaj, Iran

**Keywords:** Socio-demographic, Diabetes, Self-reporting, Validity

## Abstract

**Background:**

In this research, data of the DehPCS study were used to assess the validity of self-reported diabetes based on the reference criteria, including the history of taking oral anti-diabetic drugs, insulin injection, or high fasting blood sugar.

**Methods:**

A cross-sectional analytical study was performed on 4400 participants of the DehPCS study, aged 35–70 years. The reference criteria were oral hypoglycemic drug consumption, insulin injection, and/ or fasting blood sugar ≥126 (mg/dl). The self-reporting diabetes was investigated by well-trained interviewers before the diabetes diagnosis based on the reference criteria. The validity of self-reporting diabetes was assessed using sensitivity, specificity, as well as positive and negative predictive values. Socio-demographic correlates of self-reported agreement were examined by multinomial logistic regression.

**Results:**

Three thousand nine hundred ninety-six people participated in this study, and the participation rate was equal to 90.8%. The diabetes prevalence among the study population was 13.1% based on self-reports and 9.7% based on the reference criteria. Five hundred twenty-three participants reported diabetes, 213 (41.28%) of whom did not have it. We found a good agreement of 92.3% with an acceptable kappa value of 65.1% between self-reporting diabetes and the reference criteria. Diabetes self-reporting also guaranteed sensitivity of 78.5%, specificity of 93.9%, as well as the positive and negative predictive values of 58.7% and of 98.0%, respectively. Being female, the higher economic class, the higher body mass index (BMI), and the positive family history of diabetes increased the chance of false positive. Being male, older ages and the moderate economic class increased the chance of false positive.

**Conclusion:**

Self-reporting diabetes is identified as a relatively valid tool which could fairly determine the diabetes prevalence in epidemiological studies. It should be noted that its validity is influenced by some socio-demographic characteristics.

## Introduction

Diabetes known as the latent epidemic, is an important risk factor for cardiovascular diseases, a number of cancers, and death [1]. The latest data released by the International Diabetes Federation (IDF) show there are currently 463 million adults aged 20–79 years with diabetes, 79% of whom live in low- and middle-income countries [2]. According to the World Health Organization (WHO) in 2016, the overall prevalence of diabetes in Iran was 10.3, and 2% of the leading causes of death was due to it in 2012 [3]. The recent national study in Iran estimated the prevalence of diabetes mellitus (DM) in the range of 8.3 to 20.8% in different provinces [4].

There are several ways to evaluate diabetes, of which self-reporting by people is one of the easiest [5]. In fact, health assessment through self-reporting in large population-based studies can be an alternative to more complex processes and higher-cost methods for data collection [5]. This has led to self-reporting being used as a valid method to determine the status of diabetes in many different countries such as Japan [1, 6], China [5], the USA [7, 8], and Spain [9]. However, self-reporting may be biased so that respondents may classify themselves as ill when they are not (false positive) or do not report illness if they really are (false negative) [6].

The criterion for diagnosing diabetes is a blood sugar test or a history of using drugs reducing blood sugar. Studies, based on self-reported data provide valuable information at a lower cost, but there is still no general agreement on the reliability of such data in different cultures [10]. Different studies have found inconsistencies between the results of subjective assessment and standard diagnoses of diabetes [11, 12]. So, the accuracy and validity of diabetes estimates could be affected by self-reporting because just about half of the people with diabetes are aware of their condition [2, 4] and only one-fifth of these people have controlled their diabetes [13]. In fact, the reliability and validity of self-reporting can be related to the various socio-demographic factors, and the type of chronic diseases [10]. Individuals may not be able to diagnose their condition because they have provided incorrect information to physicians, or have forgotten or misinterpreted medical advice, or have received incorrect information from specialists [5].

There are about 40 million Kurds in the world, living in four different countries. The socio-cultural characteristics, lifestyles, eating habits, and patterns of chronic diseases of the Kurdish people differ from those of their compatriots who are not Kurds. Kurdistan is a deprived and underserved area of Iran. The Kurdish residents have remained relatively less benefited of healthcare infrastructures even after the implementation of the National Transformation Plan. This situation could affect the diagnosis and self-reporting of diabetes. So, the prevalence of type 2 diabetes in the Kurdish population is lower than that of other Iranian ethnic groups [4].

Identifying socio-demographic factors influencing validity of self-reporting diabetes can be serious for planning public health policies in more vulnerable groups. In fact, it is important both to interpret existing data and to plan future research on the diagnosis of diabetes and its consequences. Understanding the causes of inconsistencies between self-reporting diabetes and the standard criteria is a substantial basis for determining the most appropriate approach in future research programs. To this end, we used data from the Dehgolan Prospective Cohort Study (DehPCS) to assess the validity of self-reported diabetes based on the reference criteria, including the history of taking oral anti-diabetic drugs, insulin injection, or high fasting blood sugar (FBS).

## Materials and methods

### Study population

The present study was a cross-sectional analytical one which used enrollment phase data of the DehPCS. DehPCS is one of 18 prospective epidemiological cohort studies in Iran (PERSIAN), which is being performed on the population of 35–70 years old, permanent residents of Dehgolan with the aim of assessing the risk factors of common non-communicable diseases in the region. All PERSIAN sites use the same protocol to conduct the study. The questionnaires used in this study have different sections including general factors (demographic, and socioeconomic characteristics, lifestyles, environmental exposure, occupational exposure, physical activities, and personal habits), medical factors (the medical history, clinical symptoms, family health history, drug use, reproductive history, oral health, general health, anthropometry, physical exams, blood and urine analysis) and nutritional factors (the food frequency, eating habits, and supplementation). Sampling was done by a simple cluster sampling method, and 4400 eligible people were invited to participate in the study, whose participation rate was 90.8%. Out of a total of 3996 participants, 3976 had adequate information about diabetes self-reporting, blood samples, and taking medication or insulin injection, who were considered for further assessment. The design and rationale for conducting the study were previously published [14, 15].

### Data collection and measurements

In the first step, participants were invited to the study site to sign the informed consent form. Then, to collect information, they were enrolled in the online software and received a unique code. All data were collected by expert interviewers who had completed the necessary training courses according to the executive protocol. For para-clinical tests, biological samples (blood and urine) were first collected on an empty stomach. We measured the weight using the Seka scale and the height using the Seka stadiometer to the nearest 0.1 cm. The body mass index (BMI) was calculated as weight in kilograms divided by height in square meters. Blood pressure was measured using a Richter aneroid sphygmomanometer after at least 15 minutes of rest, with two measurements in the right arm at intervals of at least half an hour. The mean of the two measurements was considered as the mean of systolic and diastolic blood pressure. According to the criteria of the Joint National Committee on Prevention, Detection, Evaluation, and Treatment of High Blood Pressure (JNC 7), people with systolic blood pressure ≥ 140 mmHg, or diastolic blood pressure ≥ 90 mmHg, or people with a history of taking antihypertensive drugs are considered hypertensive. The participants’ age was considered based on their official identity cards. Education was measured based on the number of years the person had studied. The economic status was calculated based on the wealth index using the method of Multiple Correspondence Analysis (MCA) by analyzing principal components, such as durable goods, housing features, and other facilities. Individuals with a history of smoking less than 100 cigarettes during their lifetime were considered non-smokers. The use of illicit drugs was defined as the use of drugs once a week for at least 6 months, and alcohol consumption as drinking about 200 ml of beer or 45 ml of alcohol once a week for at least 6 months. The family history of diabetes was also assessed in first- and second-degree relatives. Second-degree relatives refer to people with whom we share 25% of the genome. It is noteworthy that we have collected data of self-reporting diabetes before diabetes diagnosis based on the reference criteria.

### Diabetes measurement

Diabetes self-reporting was assessed by asking the following question, “Have you ever had diabetes in the past?” People who answered yes, were asked the next question, “Who told you that you had diabetes?” All those who answered diagnosed by a physician were considered to have self-reported diabetes. The reference criterion for the diagnosis of diabetes included high fasting blood sugar (FBS) indicating diabetes, the positive history of routine insulin use and/ or oral hypoglycemic drug use. FBS was examined after 9–12 hours of fasting, and FBS ≥ 126 mg / dL (7 mmol / L) was considered as diabetes. Drug use on the day of blood sampling was assessed with the following question, “Do you routinely use anti-diabetic drugs or insulin?” If the answer was yes, the used drugs were visually evaluated.

### Statistical analysis

The validity of diabetes self-reporting was evaluated using the following criteria. Sensitivity (Se) as a proportion of people with DM who had self-reported diabetes (true positive/[true positive + false negative]), Specificity (Sp) as a proportion of people without DM who did not have self-reported diabetes or the true negative rate (true negative/[true negative + false positive], the positive predictive value (PPV) as a proportion of individuals with self-reported DM who had the reference-based DM (true positive/[true positive + false positive], the negative predictive value (NPV) as a proportion of individuals without self-reported DM who did not have reference-based DM (true negative/[true negative + false negative], the positive likelihood ratio (LR+) as Se divided by the false positive rate (FPR), the negative likelihood ratio (LR-) as the false negative rate (FNR) divided by Sp. Kappa coefficient was another calculated statistic which examined free chance concordance between two diagnostic approaches. 95% confidence intervals (CI) were calculated for all values based on the standard method for proportion. Validity was calculated overall and based on demographic, and socioeconomic characteristics, three categories of the body mass index (BMI), personal habits, and the hypertension status. Binary and multinomial logistic regression was used to examine concordance between self-reported diabetes and the reference value. To examine diagnostic characteristics of self-reported diabetes plus sex and age, we used precision-recall curve (PRC) which presents PPV against Se. The two-sided test with an alpha level of 0.05 was considered for statistical significance. All analysis was done by using Stata software version 16 (Stata Corp, College Station, TX, USA).

## Results

Out of 3976 participants with adequate information about diabetes self-reporting and the reference criteria, 2241 (56.26%) were female and 1735 (43.74%) were male. The mean age of male and female participants was 47.98 ± 8.91 and 48.78 ± 8.91 years, respectively. Most participants had a lower level of education than high school, and about 31% of them were illiterate. The mean BMI of the participants was 28.00 ± 4.58 kg/m^2^ while 32.31% of them were in the obese group with BMI ≥ 30 kg/m^2^. In terms of blood pressure, 21.50% of the participants had a systolic blood pressure ≥ 140 or a diastolic blood pressure ≥ 90. Also, 27.81% of them reported a positive history of diabetes in their first-degree relatives. Demographic characteristics and basic information of the participants have been shown in Table 1.

**Table 1:** Demographic characteristic and baseline information of the participants in DehPCS, by self-reported diabetes situation

	Total n	Diabetic n (%)	Non-diabetic n (%)	*P*-value
Gender				
Male	1735	151 (8.70)	1584 (91.30)	< 0.001
Female	2241	372 (16.60)	1869 (83.40)	
Age groups				
35–45	1773	149 (8.40)	1624 (91.60)	< 0.001
46–60	1720	261 (15.17)	1459 (84.83)	
> 60	483	113 (23.40)	370 (76.60)	
Marital status				
Married	3651	453 (12.41)	3198 (87.59)	< 0.001
Single	325	70 (21.54)	255 (78.46)	
Education years				
Illiterate	1245	262 (21.04)	983 (78.96)	< 0.001
1–5	1110	142 (12.79)	968 (87.21)	
6–12	1113	78 (7.01)	1035 (92.99)	
University	508	41 (8.07)	467 (91.93)	
Economic status				
Poorest	1344	149 (11.09)	1195 (88.91)	0.001
Moderate	1300	165 (12.69)	1135 (87.31)	
Rich	1318	208 (15.78)	1110 (84.22)	
BMI				
Normal weight	985	75 (7.61)	910 (92.39)	< 0.001
Over-weight	1698	246 (14.49)	1452 (85.51)	
Obese	1281	201 (15.69)	1080 (84.31)	
Cigarette smoking				
No smoker	3024	393 (13.00)	2631 (87.00)	< 0.001
Ex-smoker	326	68 (20.86)	258 (79.14)	
Smoker	600	61 (10.17)	539 (89.83)	
Drug use				
Yes	448	47 (10.49)	401 (89.51)	0.071
No	3502	475 (13.56)	3027 (86.44)	
Alcohol use				
Yes	481	45 (9.36)	436 (90.64)	0.008
No	3470	477 (13.75)	2993 (86.25)	
HTN				
Yes	839	182 (21.69)	657 (78.31)	< 0.001
No	3137	341 (10.87)	2796 (89.13)	
Family history of DM				
No	2436	234 (9.61)	2202 (90.39)	< 0.001
Second degree	430	63 (14.65)	367 (85.35)	
First degree	1106	226 (20.43)	880 (79.57)	

**Fig. 1:**
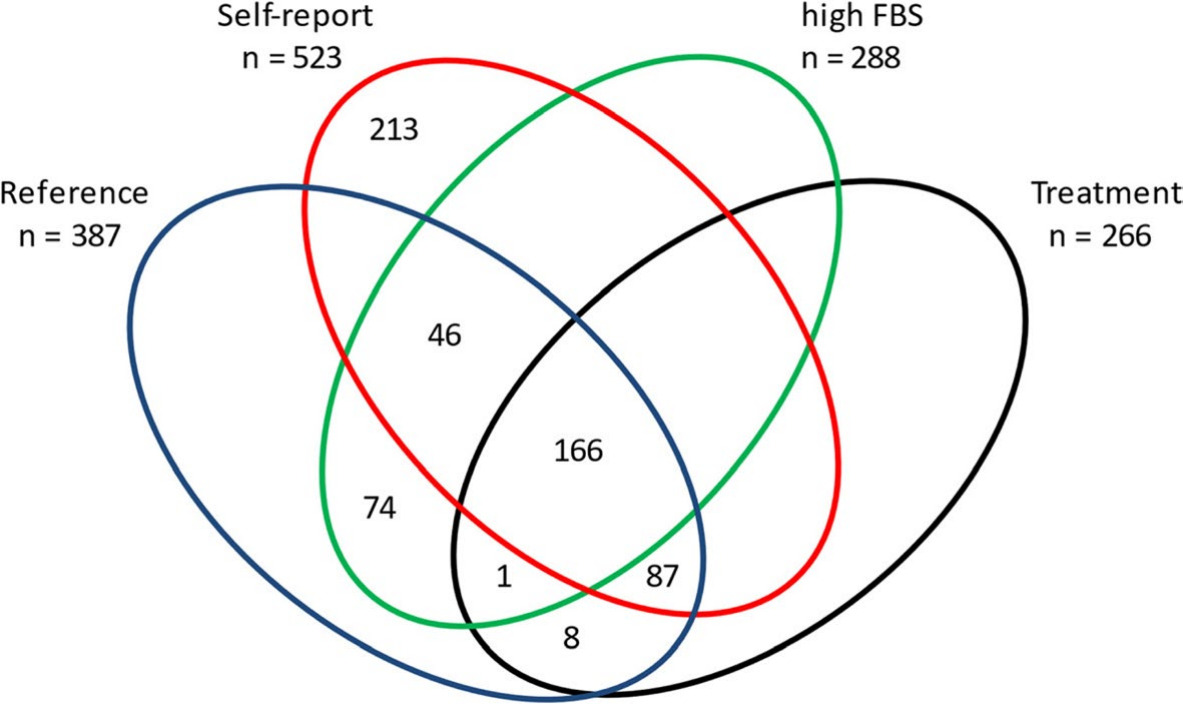
Frequency overlap of self-reported diabetes and diabetes measured by reference criteria (high FBS + treatment)

The diabetes prevalence in the study population was 13.1, and 9.7% based on self-reporting, and the reference criteria, respectively. Out of the 523 participants who reported diabetes, 213 (41.28%) did not have diabetes according to the reference criteria. One hundred sixty-seven people of the participants treated for diabetes (63.74%), had poorly controlled diabetes, and 83 people of ones with diabetes (with high FBS or treated with drugs or insulin (21.50%)), did not know they had it (Fig. 1).

**Fig. 2:**
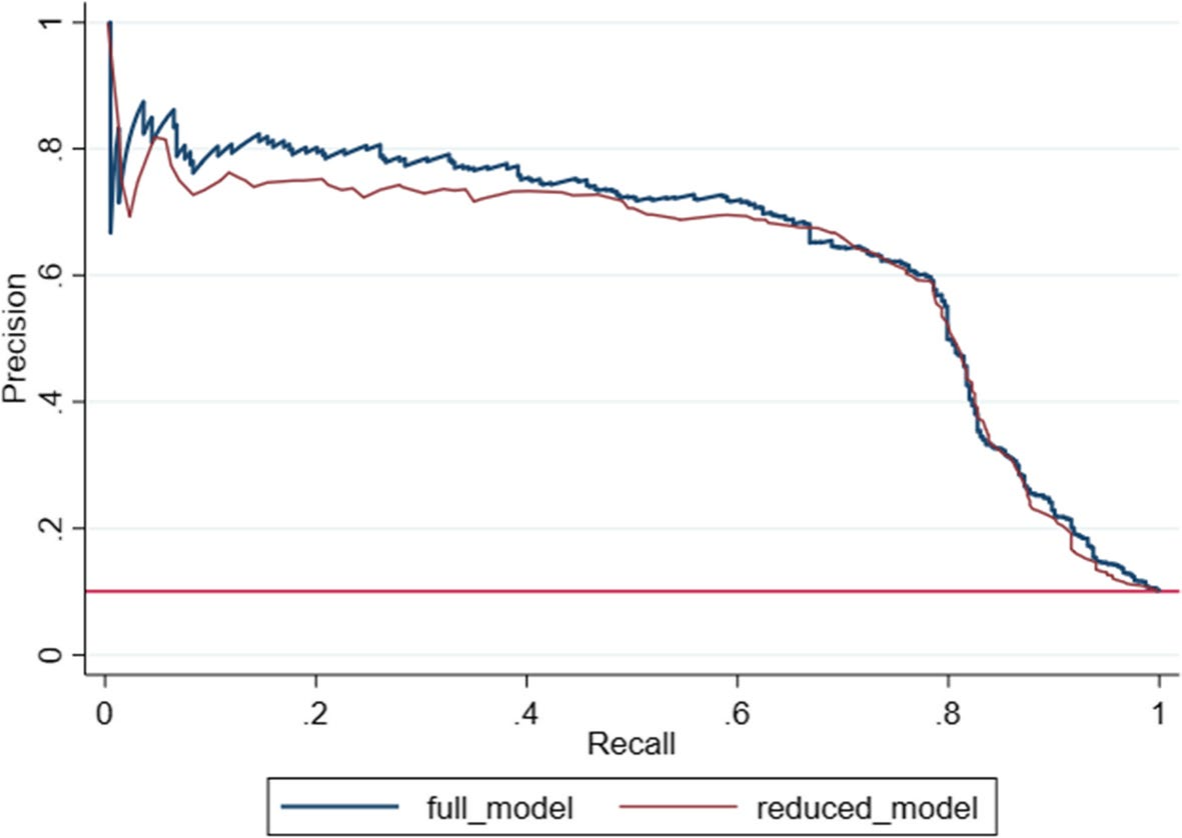
Diagnostic characteristics of reduced model (Self-reported diabetes, Sex, Age) in comparison with full model (Self-reported diabetes, sex, age, marital status, economic status, BMI, smoking status, alcohol use, HTN, and family history of HTN)

Table 2 shows the validation of diabetes self-reporting based on demographic, socioeconomic, and some individual variables. The percentage of general agreement and agreement based on kappa statistics was 92.3 and 65.1%, respectively. The estimated value of kappa statistic varied between 45.5 and 81.1% based on the study participants’ characteristics. In general, the kappa agreement was higher in men, older groups, people with the poor economic status, people with normal weight, ex-smokers, and people with high blood pressure. The overall Se and Sp were 78.5 and 93.9%, respectively. Se increased with age while it decreased with weight. The total PPV and NPV were 58.7 and 98.0%, respectively. Unlike Se, PPV was significantly higher in men than in women, and with age, it increased by more than 38% so that in the age group over 60 years, it reached more than 72%. Also, PPV was higher among people with hypertension and those with a positive family history of diabetes. Figure 2 shows the PRC. The area under the curve was 64.24% for the full model and 61.60% for the reduced model.

**Table 2:** Validity of self-reported diabetes using reference criteria in DehPCS

	Concordance% (CI)	Kappa% (CI)	Sensitivity% (CI)	Specificity% (CI)	+LR	-LR	PPV% (CI)	NPV% (CI)
Overall	92.3 (90.9–93.7)	65.1 (61.8–68.3)	78.5 (74.1–82.5)	93.9 (93.0–94.6)	12.78 (11.11–14.70)	0.23 (0.19–0.28)	58.7 (54.3–63.0)	98.0 (97.4–98.4)
Gender								
Female	90.2 (88.3–92.1)	61.6 (57.3–65.9)	84.2 (78.9–88.6)	91.3 (90.0–92.5)	9.71 (8.34–11.31)	0.17 (0.13–0.23)	53.8 (48.7–59.0)	98.0 (97.3–98.6)
Male	95.1 (93.0–97.2)	72.2 (66.2–76.3)	76.1 (68.1–82.9)	97.1 (96.2–97.9)	26.42 (19.57–36.65)	0.25 (0.18–0.33)	70.7 (62.7–77.8)	97.3 (96.4–98.1)
Age groups								
35–45	93.2 (91.0–95.2)	45.5 (40.8–50.2)	69.9 (58.0–80.1)	94.2 (93.0–95.3)	12.12 (9.49–15.47)	0.32 (0.23–0.45)	34.2 (26.7–42.4)	98.6 (98.0–99.1)
46–60	92.5 (90.3–94.7)	70.8 (65.8–75.8)	85.0 (79.3–89.6)	94.0 (92.7–95.2)	14.20 (11.54–17.47)	0.16 (0.11–0.22)	65.1 (59.0–70.9)	97.9 (97.1–98.6)
> 60	88.7 (84.5–92.9)	70.5 (61.6–79.4)	82.0 (73.1–89.0)	91.9 (88.7–94.4)	10.13 (7.14–14.37)	0.20 (0.13–0.30)	72.6 (63.4–80.5)	95.1 (92.4–97.1)
Marital status								
Married	92.9 (91.4–94.3)	66.2 (62.8–69.7)	81.4 (76.8–85.5)	94.4 (93.6–95.2)	14.54 (12.53–16.88)	0.20 (0.16–0.25)	58.9 (54.3–63.5)	98.1 (97.6–98.5)
Single	85.9 (80.4–91.3)	55.3 (51.9–58.7)	79.5 (64.7–90.2)	87.5 (83.1–91.2)	6.39 (4.53–9.01)	0.23 (0.13–0.42)	50.0 (37.8–62.2)	96.5 (93.4–98.4)
Education years								
Illiterate	89.2 (86.6–91.8)	66.4 (60.6–72.2)	86.4 (80.6–91.0)	90.3 (88.4–92.0)	8.90 (7.34–10.79)	0.15 (0.10–0.22)	60.7 (54.5–66.6)	97.5 (96.3–98.3)
1–5	91.9 (89.2–94.6)	61.6 (55.4–67.8)	79.2 (69.7–86.8)	93.5 (91.8–94.9)	12.16 (9.43–15.69)	0.22 (0.15–0.33)	53.5 (45.0–61.9)	97.9 (96.8–98.7)
6–12	94.9 (92.3–97.5)	51.5 (55.2–67.8)	69.8 (57.0–80.8)	96.8 (95.5–97.7)	21.57 (14.92–31.17)	0.31 (0.21–0.45)	56.4 (44.7–67.6)	98.2 (97.1–98.9)
university	95.1 (91.4–98.8)	65.1 (55.9–74.2)	79.3 (60.3–92.0)	96.2 (94.1–97.8)	21.11 (12.93–34.45)	0.21 (0.11–0.44)	56.1 (39.7–71.5)	98.7 (97.2–99.5)
Economic status								
Poor	94.7 (92.4–97.0)	72.3 (66.7–78.0)	87.9 (80.1–93.4)	95.6 (94.3–96.6)	19.76 (15.12–25.83)	0.13 (0.08–0.21)	63.1 (54.8–70.8)	98.9 (98.1–99.4)
Moderate	91.3 (88.7–94.0)	60.4 (54.6–66.2)	75.6 (66.9–83.0)	93.6 (92.1–95.0)	11.91 (9.35–15.16)	0.26 (0.19–0.36)	54.5 (46.6–62.3)	97.4 (96.4–98.3)
Rich	90.7 (88.3–93.1)	62.9 (57.3–68.6)	80.7 (73.3–86.8)	92.2 (90.6–93.7)	10.40 (8.41–12.87)	0.21 (0.15–0.29)	56.3 (49.2–63.1)	97.5 (96.4–98.3)
BMI								
Normal weight	95.9 (93.1–98.6)	70.3 (63.6–76.9)	85.2 (72.9–93.4)	96.9 (95.6–97.9)	27.35 (18.79–39.79)	0.15 (0.08–0.29)	61.3 (49.4–72.4)	99.1 (98.3–99.6)
Over-weight	92.2 (90.1–94.4)	68.5 (63.5–73.5)	82.9 (76.7–88.0)	94.0 (92.7–95.1)	13.76 (11.16 16.97)	0.18 (0.13–0.25)	63.0 (56.6–69.1)	97.8 (96.9–98.5)
Obese	89.7 (87.2–92.3)	57.8 (52.1–63.5)	77.7 (69.6–84.5)	91.3 (89.5–92.9)	8.94 (7.26–11.02)	0.24 (0.18–0.34)	50.2 (43.1–57.4)	97.3 (96.2–98.2)
Cigarette smoking								
No smoker	92.0 (90.4–93.6)	62.3 (58.5–66.0)	80.6 (75.3–85.2)	93.4 (92.5–94.3)	12.30 (10.55–14.33)	0.21 (0.16–0.27)	53.9 (48.9–59.0)	98.1 (97.5–98.6)
Ex-smoker	93.7 (89.1–98.4)	81.1 (69.6–92.6)	94.4 (84.6–98.8)	93.8 (90.2–96.3)	15.11 (9.49–24.05)	0.06 (0.02–0.18)	75.0 (63.0–84.7)	98.8 (96.6–99.8)
Smoker	92.9 (89.4–96.5)	63.3 (54.8–71.9)	70.4 (56.4–82.0)	95.8 (93.7–97.3)	16.71 (10.80–25.83)	0.31 (0.20–0.47)	62.3 (49.0–74.4)	97.0 (95.2–98.3)
Drug use								
No	92.0 (90.5–93.5)	64.4 (60.9–67.9)	81.6 (77.0–85.6)	93.5 (92.6–94.4)	12.62 (10.95–14.54)	0.20 (0.16–0.25)	56.8 (52.3–61.3)	98.0 (97.4–98.5)
Yes	94.2 (90.3–98.1)	69.7 (59.9–79.5)	77.5 (61.5–89.2)	96.1 (93.7–97.7)	19.76 (11.89–32.86)	0.23 (0.13–0.42)	66.0 (50.7–79.1)	97.8 (95.8–99.0)
Alcohol use								
No	92.0 (90.5–93.5)	65.2 (61.7–68.7)	81.3 (76.7–85.3)	93.6 (92.7–94.5)	12.78 (11.07–14.75)	0.20 (0.16–0.25)	58.3 (53.7–62.7)	97.9 (97.3–98.3)
Yes	94.0 (90.1–98.0)	61.7 (52.3–71.0)	79.3 (60.3–92.0)	95.1 (92.7–96.9)	16.29 (10.41–25.50)	0.22 (0.11–0.44)	51.1 (35.8–66.3)	98.6 (97.0–99.5)
HTN								
No	92.7 (91.2–94.3)	59.6 (56.0–63.3)	77.9)71.9–83.2(	94.2 (93.3–95.1)	13.52 (11.49–15.91)	0.23 (0.18–0.30)	50.7 (45.3–56.2)	98.2 (97.7–98.7)
Yes	90.7 (87.4–93.9)	73.2 (65.9–80.4)	86.0 (79.4–91.1)	92.3 (90.1–94.2)	11.18 (8.56–14.60)	0.15 (0.10–0.23)	70.9 (63.7–77.4)	96.8 (95.2–98.0)
Family history of DM								
No	93.0 (91.2–94.8)	59.4 (55.2–63.6)	72.8 (65.6–79.3)	95.2 (94.3–96.1)	15.26 (12.43–18.74)	0.29 (0.22–0.36)	53.8 (47.2–60.4)	97.9 (97.2–98.4)
Second degree	94.1 (90.0–98.3)	74.3 (64.4–84.2)	95.0 (83.1–99.4)	93.6 (90.7–95.8)	14.82 (10.08–21.80)	0.05 (0.01–0.21)	60.3 (47.2–72.4)	99.5 (98.0–99.9)
First degree	90.0 (87.3–92.7)	67.8 (61.6–73.9)	86.8 (80.5–91.6)	90.7 (88.7–92.5)	9.34 (7.59–11.50)	0.15 (0.10–0.22)	61.1 (54.4–67.5)	97.6 (96.4–98.5)

According to Table 3, in multivariate analysis, independent factors increasing inconsistencies between diabetes self-reporting and the reference value included the female gender, celibacy, moderate to high economic status, higher BMI, and having a first-degree relative with diabetes. The results of multinomial logistic regression suggested females, single people, those in the upper economic class, people with higher BMI, and those having a first-degree relative with diabetes were more likely to falsely report diabetes (FP). Conversely, the probability of false reports of not having diabetes was higher in men, older people, and those in the lower economic class. However, the female gender, older ages, higher BMI, previous history of smoking, high blood pressure, and positive family history of diabetes significantly increased the true reports of diabetes mellitus (TP) compared to those of non-diabetes (TN).

**Table 3:** Univariable and multivariable analysis of factors affecting validity of self-reported diabetes in DehPCS

Variables	disagreement self-reported^1^		false positive self-reported^2^		false negative self-reported^2^		true positive self-reported^2^	
	Crud OR (CI)	Adjusted OR	Crud OR	Adjusted OR	Crud OR	Adjusted OR	Crud OR	Adjusted OR
Gender (reference: female)	0.49 (0.38–0.64(^c^	0.69 (0.52–0.92)^a^	0.31 (0.22–0.43)^c^	0.33 (0.52–0.52)^c^	1.15 (0.75–1.78)	1.86 (1.05–3.31)^a^	0.64 (0.50–0.81)^c^	0.66 (0.47–0.91)^a^
Age	1.02 (1.00–1.03)^b^	1.01 (1.00–1.03)	1.02 (1.01–1.04)^a^	1.02 (1.00–1.04)	1.05 (1.02–1.07)^c^	1.04 (1.01–1.07)^a^	1.08 (1.07–1.09)^c^	1.08 (1.06–1.10)^c^
Marital status (reference: married)	2.10 (1.49–2.96)^c^	1.54 (1.06–2.24)^a^	2.41 (1.63–3.55)^c^	1.52 (1.00–2.33)^a^	1.74 (0.88–3.41)	1.59 (0.76–3.34)	1.66 (1.14–2.41)^b^	0.80 (0.52–1.23)
Education years	0.94 (0.92–0.96)^c^	0.97 (0.95–1.00)	0.92 (0.89–0.94)^c^	0.96 (0.93–1.01)	0.97 (0.93–1.01)	1.04 (0.97–1.10)	0.91 (0.88–0.93)^c^	0.98 (0.94–1.01)
Economic status (reference: poor)								
Moderate	1.66 (1.21–2.27)^c^	1.62 (1.18–2.22)^b^	1.56 (1.08–2.25)^a^	1.43 (0.98–2.08)	1.97 (1.10–3.51)^a^	1.95 (1.08–3.53)^a^	1.02 (0.75–1.37)	0.82 (0.59–1.13)
Rich	1.86 (1.37–2.53)^c^	1.60 (1.15–2.20)^b^	1.89 (1.33–2.70)^c^	1.46 (1.00–2.13)^a^	1.95 (1.09–3.50)^a^	1.71 (0.92–3.17)	1.35 (1.02–1.79)^a^	0.81 (0.59–1.12)
BMI	1.08 (1.05–1.10)^c^	1.06 (1.04–1.09)^c^	1.08 (1.05–1.11)^c^	1.05 (1.02–1.08)^c^	1.08 (1.04–1.13)^c^	1.11 (1.07–1.16)^c^	1.05 (1.03–1.08)^c^	1.03 (1.00–1.06)^a^
Cigarette smoking (reference: no smoker)								
Ex-smoker	0.87 (0.55–1.37)	–	0.94 (0.55–1.59)	1.40 (0.78–2.50)	1.04 (0.44–2.43)	0.74 (0.30–1.83)	2.45 (1.75–3.41)^c^	2.12 (1.44–3.14)^c^
Smoker	0.88 (0.62–1.24)	–	0.65 (0.42–1.02)	1.30 (0.76–2.21)	1.51 (0.88–2.58)	1.46 (0.77–2.76)	0.88 (0.62–1.26)	1.22 (0.79–1.87)
Drug use (reference: no)	0.70 (0.46–1.06)	–	0.57 (0.33–0.97)^a^	0.82 (0.42–1.61)	1.02 (0.52–2.00)	0.91 (0.40–2.05)	0.85 (0.58–1.25)	0.66 (0.39–1.12)
Alcohol use (reference: no)	0.81 (0.54–1.19)	–	0.75 (0.47–1.19)	1.41 (0.83–2.41)	0.83 (0.41–1.68)	0.66 (0.31–1.41)	0.56 (0.36–0.88)^a^	0.65 (0.40–1.06)
HTN (reference: no)	1.36 (1.04–1.78)^a^	1.02 (0.77–1.37)	1.39 (1.00–1.92)^a^	0.98 (0.68–1.42)	2.10 (1.25–3.22)^b^	1.18 (0.69–2.02)	3.27 (2.56–4.17)^c^	1.79 (1.35–2.37)^c^
Family history of DM (reference: no)								
Second degree	0.88 (0.57–1.36)	0.92 (0.59–1.43)	1.28 (0.81–2.05)	1.32 (0.82–2.12)	0.22 (0.05–0.89)^a^	0.27 (0.07–1.14)	1.78 (1.21–2.59)^b^	2.53 (1.69–3.81)^c^
First degree	1.60 (1.24–2.06) c	1.57 (1.21–2.04)^c^	2.03 (1.51–2.73)^c^	1.95 (1.44–2.65)^c^	1.25 (0.78–2.00)	1.48 (0.91–2.41)	2.76 (2.14–3.56)^c^	3.43 (2.61–4.51)^c^

^1^ logistic regression, ^2^ multinomial logistic regression with true negative self-reported diabetes as reference category, adjusted variables: Cigarette smoking, Drug use, Alcohol use, ^a^ *p*-value < 0.05, ^b^ *p*-value < 0.01, ^c^ *p*-value < 0.001

## Discussion

In this study aimed to assess the validity of self-reporting diabetes in a large Kurdish population, we found self-reported diabetes had a moderate sensitivity of 78.5%, a high specificity of 93.9%, a fairly good positive predictive value of 58.7%, and a high negative predictive value of 98.0%. The agreement between self-reporting diabetes and the reference criteria was fairly good with Kappa of 65.1% and concordance of 92.3%. Besides, we showed that the participants’ demographic, anthropometric, and habitual features largely influenced the accuracy of self-reported diabetes. In this case, being female, increase in age, increase in BMI, being an ex-smoker, having HTN, and the positive family history of diabetes caused an increase in the odds of the true positive rate in diabetes self-reports. We found 31% of diabetic participants (120 out of a total of 386) were not under any medication for diabetes. The previous reports on this issue showed almost the same statistics [16, 17]; however, we demonstrated an updated validation of diabetes self-reports among a large Kurdish population of Iran.

The epidemiological surveys commonly apply either self-reports or medical records of chronic diseases to estimate their incidence or prevalence [18]. Among chronic diseases, self-reports of diabetes were identified to be more accurate with a higher level of agreement [19–21]. Our findings on the accuracy of self-reported diabetes were in line with the recent similar studies showing the sensitivity of 75–79.3% and specificity of 95.8–98.4% [17, 22]. However, older previous studies showed lower sensitivity of 61.5–69.7% for diabetes self-reports [21, 23]. This increasing trend in the accuracy of diabetes self-reports can be explained by the increase in awareness of the society and the development of the health care system over time [24]. Meanwhile, the difference in this accuracy over time can be due to the different demographic features of the studied population since we, similar to previous studies, revealed that the accuracy of diabetes self-reports was largely dependent on the baseline characteristics of study participants [5, 17, 21].

The results of the multivariable analysis showed that women were more likely to have a disagreement of self-reported diabetes with the reference, higher false positive and true positive rates and lower false negative rates than men. One explanation for this finding is that women take better self-care behaviors and use more health care services [25]. Moreover, women take more attention to their dietary consumption. In this instance, they tend to count daily intake of carbohydrates and consume less fat [26]. Thus, they are more likely to find themselves in diabetic conditions and report more true positives and false positives. We also found that increment in the study participants’ age was associated with higher odds of true positive and false negative rates of self-reported diabetes. The higher false negative self-reports in older participants can be due to a recall bias because of Alzheimer’s disease or age-related memory loss [27] and higher true positive self-reports among older individuals can be due to more health care delivery and more opportunities to undergo blood sugar testing in this population [25]. We also observed that increment in BMI was associated with higher odds of the discordance between diabetes self-reports and the reference criteria, higher true positives, false negatives, and false positives of self-reported diabetes. In the previous studies in line with this study, obesity, as well as an increase in BMI resulted in higher odds of diabetes development in this population and consequently higher true positive and false negative rates [17, 21, 28]. This finding can be attributed to insulin resistance conditions in obesity as well as poor self-care of overweight and obese individuals [29, 30].

In this study, the results showed the education level was not significantly related to self-reported disagreement, self-reported false positive, self-reported false negative, and self-reported true positive. These results confirmed the findings of the study of M. Huerta et al. which showed that in patients with low education, the percentage of false negative and false positive of self-reporting was high but it was not statistically significant [21].

In this study, participants with HTN were more likely to truly report their diabetes. This finding can be due to better monitoring of other metabolic syndrome risk factors in this population and higher awareness about their health. In line with previous studies, we observed no significant change in the odds of false negative and false positive rates among populations with HTN [17].

The positive family history of diabetes, particularly from the first-degree relatives, showed a high level of discordance in diabetes self-reports. In this instance, participants with a positive family history were more likely to develop diabetes and this issue explains higher true positive rates of diabetes self-reports in this group. Besides, similar to previous studies, participants with the positive family history tend to more frequently report diabetes, which leads to higher false positive rates [17, 31].

### Strengths and limitations

This study had several strengths worth to be stated. It was a large population-based survey derived from the PERSIAN cohort of Iran and had a low risk of attrition bias with a high response rate (91%) of enrolled residents of Dehgolan, the Kurdish region of Iran. Thus, we could generalize our findings to the whole Kurdish population of Iran. This study also had several limitations worth to be discussed. It examined the self-reported prevalent diabetes; thus, this validation could not be applied for the studies investigating incident diabetes. As stated, this validation was conducted in the west part of Iran and due to the racial, ethnic, and socio-cultural diversity of other regions of Iran, we require further validation to determine the accuracy of self-reported diabetes in other populations and to elucidate the impact of socio-cultural nature of each region on the accuracy and discordance of self-reported diabetes. Diabetes is a well-known chronic disease with a standard definition criterion. We did not use glycosylated hemoglobin alongside the standard definition and it should be considered when generalizing results.

## Conclusion

We found self-reported diabetes with moderate sensitivity indicating high awareness of the general Kurdish population of Iran about their diabetic status, high specificity, fairly good PPV, and very high NPV reflecting good accuracy of self-reported diabetes for detecting diabetes in this population. We also found good agreement between self-reported diabetes and the reference criteria. Thus, diabetes self-reporting could be used as a relatively valid tool to identify diabetes prevalence in future epidemiological studies on the Kurdish population of Iran. Besides, we revealed individuals’ socio-demographic and habitual characteristics largely affected this validity and they should be considered to warrant more accurate estimation.

## Data Availability

The data of this study are available on request from the corresponding author (FM). The data are not publicly available due to privacy and ethical restrictions.

## Abbreviations

Se: Sensitivity
Sp: Specificity
NPV: Negative Predict Value
PPV: Positive Predict Value
HTN: Hypertension
BMI: Body Mass Index
PRC: Precision-Recall Curve
FP: False Positive
TP: True Positive
FN: False Negative
TN: True Negative
FBS: Fasting Blood Sugar
DehPCS: The Dehgolan Prospective Cohort Study
PERSIAN: Prospective Epidemiological Cohort Studies in Iran
JNC 7: Joint National Committee on Prevention, Detection, Evaluation, and Treatment of High Blood Pressure
MCA: Multiple Correspondence Analysis
